# Mixed‐Organic‐Cation Tin Iodide for Lead‐Free Perovskite Solar Cells with an Efficiency of 8.12%

**DOI:** 10.1002/advs.201700204

**Published:** 2017-07-14

**Authors:** Ziran Zhao, Feidan Gu, Yunlong Li, Weihai Sun, Senyun Ye, Haixia Rao, Zhiwei Liu, Zuqiang Bian, Chunhui Huang

**Affiliations:** ^1^ Beijing National Laboratory for Molecular Sciences State Key Laboratory of Rare Earth Materials Chemistry and Applications College of Chemistry and Molecular Engineering Peking University Beijing 100871 P. R. China; ^2^ Institute of Modern Optics and State Key Laboratory for Artificial Microstructure and Mesoscopic Physics School of Physics Peking University Beijing 100871 P. R. China

**Keywords:** cation mixing, lead‐free, perovskite solar cells, power‐conversion efficiency, tin‐based perovskites

## Abstract

In this work, a fully tin‐based, mixed‐organic‐cation perovskite absorber (FA)*_x_*(MA)_1−_ *_x_*SnI_3_ (FA = NH_2_CH = NH_2_^+^, MA = CH_3_NH_3_^+^) for lead‐free perovskite solar cells (PSCs) with inverted structure is presented. By optimizing the ratio of FA and MA cations, a maximum power conversion efficiency of 8.12% is achieved for the (FA)_0.75_(MA)_0.25_SnI_3_‐based device along with a high open‐circuit voltage of 0.61 V, which originates from improved perovskite film morphology and inhibits recombination process in the device. The cation‐mixing approach proves to be a facile method for the efficiency enhancement of tin‐based PSCs.

## 1 Introduction

Over the past few years, perovskite solar cells (PSCs) have emerged as a rising star in the field of solar cells. The power conversion efficiency (PCE) of PSCs has been boosted from an initial 3.8%^1^ to an astounding 22.1%,^2^ which benefitted from the merits of lead (Pb) halide materials (APbX_3_, A = Cs^+^, CH_3_NH_3_^+^, NH_2_CH = NH_2_^+^, etc.; X = I^−^, Br^−^, Cl^−^), such as high absorption coefficient,^3^, ^4^ small exciton binding energy,^5^, ^6^ and long carrier diffusion lengths.^7^, ^8^ However, the bandgaps of Pb‐based perovskites are normally larger than 1.4 eV, which is not ideal for approaching the Shockley–Queisser limit (33%) that corresponds to a bandgap of 1.34 eV.^9^, ^10^ Tin (Sn)‐based perovskites, being structurally similar to their Pb‐based counterparts, have attracted wide attention recently. With slightly narrower bandgaps (≈1.3 eV) compared to their Pb‐based counterparts,^11^, ^12^ Sn‐based perovskites promise higher short‐circuit current densities (*J*_sc_) as well as a theoretical PCE limit that is very close to 33%. However, the efficiency of Sn‐based PSCs, though outperforming other Pb‐free candidates, still lags behind Pb‐based ones, which can be attributed to several reasons. The easy oxidation of Sn^2+^ to Sn^4+^, due to the lack of inert pair effect as in Pb^2+^, causes severe device deterioration in ambient environment. The low formation energy of Sn vacancies (*V*_Sn_) often results in high‐doped hole concentrations (i.e., up to 10^19^ cm^−3^ in polycrystalline films) in Sn‐based perovskites,^13^, ^14^ leading to severe carrier recombination in the solar cells. Besides, the rapid reaction between SnI_2_ and organic ammonium salts amplifies the difficulty in controlling film morphology via solution processing.^15^ Moreover, the functional layers (i.e., hole‐/electron‐transport layer) inherited from Pb‐based PSCs may bring about poor energy level alignment and device instability in Sn‐based devices, which further limit their performance.

Since 2014, some important progress has been made in this field. For example, SnF_2_, as an additive, has been found to effectively suppress the doped hole density in Sn‐based perovskites and improve device stability and reproducibility.^16^, ^17^ The use of dimethyl sulfoxide (DMSO) as the precursor solvent has proven to be crucial in acquiring homogeneous films, as it forms a SnI_2_·3DMSO intermediate phase that retards the rapid crystallization of Sn‐based perovskites.^18^ Seo and co‐workers employed a SnF_2_‐pyrazine complex to improve the morphology of FASnI_3_ films and obtained a PCE of 4.8%.^19^ There are also reports on adopting more suitable device structures for Sn‐based PSCs. Yan and co‐workers adopted an inverted structure for FASnI_3_‐based PSCs, and boosted the PCE to 6.22% by employing SnF_2_ additives and diethyl ether dripping in the fabrication.^20^ Recently, Ke et al. employed a TiO_2_–ZnS cascade electron transport layer to match the high conduction band minima (CBM) of FASnI_3_ and enhanced the open‐circuit voltage (*V*_oc_) of the device.^21^

Composition engineering has proven to be an effective way to tailor the properties of perovskites and enhance the performance of PSCs due to the easily adjusted composition of these materials. The mixing of the monovalent cations is one of the most commonly employed methods in the composition engineering of Pb‐based perovskites^22^, ^23^, ^24^ and Pb/Sn‐based binary perovskites,^25^, ^26^, ^27^ which combines the merits of perovskites with different cations. To the best of our knowledge, there has been no report by now on the composition engineering of purely Sn‐based perovskites.

In this article, we report the use of composite perovskites (FA)*_x_*(MA)_1−_ *_x_*SnI_3_ (FA = NH_2_CH = NH_2_^+^, MA = CH_3_NH_3_^+^) as the light‐harvesting layers in Pb‐free PSCs. SnF_2_ is employed as an additive in the perovskite precursors with DMSO as the solvent. The (FA)*_x_*(MA)_1_ *_−x_*SnI_3_ films are deposited via a one‐step method, and a series of experiments is conducted to investigate the impact of organic‐cation mixing on their structural and optical properties. To study the performance of these perovskites in solar cells, devices with the structure of indium tin oxide (ITO)/poly(3,4‐ethylenedioxythiophene) polystyrene sulfonate (PEDOT:PSS)/perovskite/C_60_/bathocuproine (BCP)/silver (Ag) were fabricated. We demonstrate that cation mixing can effectively enhance the photovoltaic performance, especially the *V*_oc_, of Sn‐based PSCs via improving the perovskite film morphology and inhibiting carrier recombination in the devices. Consequently, we achieved an optimal PCE of 8.12% for the device based on (FA)_0.75_(MA)_0.25_SnI_3_ with 10 mol% SnF_2_ additive, along with a *V*_oc_ of 0.61 V, a *J*_sc_ of 21.2 mA cm^−2^, and a fill factor (FF) of 62.7% under forward scan mode. The (FA)_0.75_(MA)_0.25_SnI_3_‐based device also exhibited high reproducibility with an average PCE of 7.29% ± 0.55% for 30 devices.

## 2 Result and Discussion

**Figure 1:**
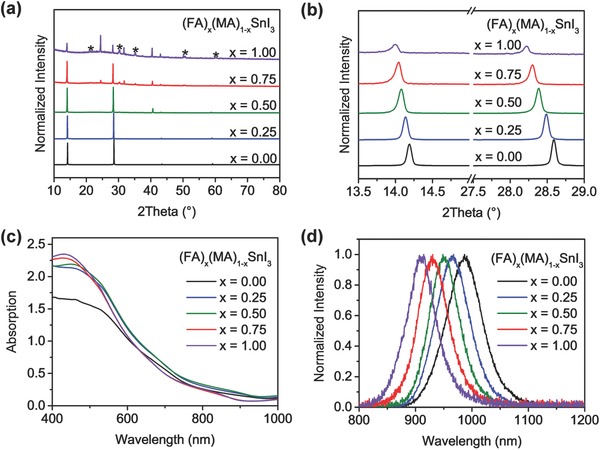
a) XRD patterns of (FA)*_x_*(MA)_1_ *_−x_*SnI_3_ (*x* = 0.00, 0.25, 0.50, 0.75, and 1.00) films deposited via one‐step method on ITO/PEDOT:PSS substrates. The peak intensity is normalized since it drops substantially as the FA content is increased. The asterisks (*) mark the diffraction peaks of ITO substrates. b) Zoomed‐in XRD patterns in the region 13.5°–29.0°. c) Absorption spectra of the different perovskite films on quartz substrates. d) Normalized emissions of the different perovskite films on quartz substrates.

The (FA)*_x_*(MA)_1_ *_−x_*SnI_3_ (*x* = 0.00, 0.25, 0.50, 0.75, and 1.00) films are deposited via a one‐step procedure, where chlorobenzene is added as the antisolvent during the spin‐coating process to induce fast crystallization of the perovskites, while DMSO was used as the solvent of the Sn‐based perovskite precursor for the formation of high‐quality films. Meanwhile, SnF_2_ was included in the precursor as an additive, as it is reported to suppress the doped hole density in Sn‐based perovskites.^16^, ^17^ To investigate the intercalation of FA and MA cations in our mixed‐cation perovskites, we measured the X‐ray diffraction (XRD) patterns of these perovskite films on ITO/PEDOT:PSS substrates (**Figure** **1**a). The main peaks are located at around 14° and 28° for MASnI_3_ and the mixed‐cation perovskites, which can be ascribed to the (101) and (202) lattice planes. For the FASnI_3_ film, the (101) and (202) peaks become much weaker, while the (201) peak at around 24° becomes the highest. Only one peak is observed for each lattice planes for the mixed‐cation perovskites, indicating that the FA and MA cations are evenly distributed in the lattices rather than forming phases of different species. Moreover, the diffraction angles decrease continuously for the (101) and (202) peaks as FA content increases (Figure 1b). The peak shift toward lower diffraction angles suggests the expansion in the lattice parameters of (FA)*_x_*(MA)_1_ *_−x_*SnI_3_, which is probably caused by the gradual replacement of the smaller MA cations by the larger FA cations.

**Figure 2:**
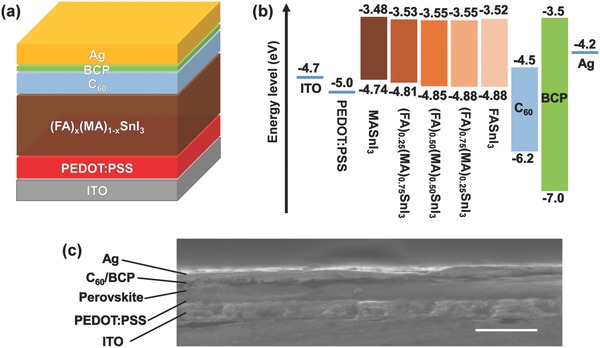
a) Schematic illustration of the device structure. b) Band alignment diagram. c) Cross‐sectional scanning electron microscope (SEM) image of a completed device (scale bar: 500 nm).

The impact of cation mixing on the optical properties of the perovskites was then investigated. We measured the absorption spectra of the (FA)*_x_*(MA)_1_ *_−x_*SnI_3_ films on quartz substrates (Figure 1c). The absorption onsets of the Sn‐based perovskites are quite indistinct, which may be ascribed to the formation of sub‐bandgap states as a result of the oxidation of Sn^2+^ during the measurement in air,^12^, ^27^ making it difficult to determine their optical bandgaps. Therefore, we obtained the steady‐state photoluminescent (PL) spectra of the encapsulated perovskite films (Figure 1d) to access their bandgaps, which calculated from the PL emission peaks are 1.26, 1.28, 1.30, 1.33, and 1.36 eV for *x* = 0.00, 0.25, 0.50, 0.75, and 1.00, respectively, showing a growing trend as the FA contents increases. The valence band maxima (VBM) of these perovskites were deduced from their ionization potentials obtained by photoelectron spectroscopy (Figure S1, Supporting Information), and the CBM were calculated accordingly form their bandgaps and VBM values (summarized in **Figure** **2**b). We noticed that some studies have reported the VBM of FAPbI_3_ to be −5.9 to −6.0 eV,^20^, ^28^ which is much lower than our result (−4.88 eV). Since Sn‐based perovskites are more prone to oxidation, their VBM are expected to be higher than that of their Pb‐based counterparts (≈−5.4 eV). We measured the VBM of FASnI_3_ at different times after the film is exposed to air (Figure S2, Supporting Information), which increased continuously and reached −5.71 eV after 99 min. XRD patterns of the perovskite films measured in ambient environment show positive shifts for the (101) and (202) peaks along with time (Figure S3, Supporting Information), which can be attributed to the oxidation of Sn^2+^ to Sn^4+^.^29^ Therefore, we speculate that oxidation may cause the lowering of their VBM and that the −5.9 to −6.0 eV results may be obtained after the FASnI_3_ film is heavily oxidized. It is also worth noting that the VBM values of many papers regarding FASnI_3_ or MASnI_3_ have reported their VBM to be around −4.8 eV,^12^, ^15^, ^21^, ^30^ which are in good agreement with our results. We herein emphasize the importance to minimize oxidation during the VBM measurements of Sn‐based perovskites.

The schematic device structure and the band alignment diagram of the (FA)*_x_*(MA)_1_ *_−x_*SnI_3_‐based PSCs are illustrated in Figure 2. Our devices consist of an ITO transparent electrode, a PEDOT:PSS hole‐transport layer (48 nm), a perovskite layer (144 ± 6, 149 ± 6, 151 ± 6, 151 ± 6, and 150 ± 6 nm for *x* = 0.00, 0.25, 0.50, 0.75, and 1.00, respectively) as the light absorber, an electron‐transport C_60_ layer (50 nm), a hole‐blocking BCP layer (8 nm), and a Ag back electrode (100 nm). As suggested by previous research, the work function of PEDOT:PSS can be shifted to 4.7–4.8 eV after the perovskite layer is deposited via one‐step method, since the surface of PEDOT:PSS is reduced by the organic ammonium salts in the perovskites.^31^, ^32^ Therefore, it is plausible to speculate that the work function of PEDOT:PSS is more or less lowered during the perovskite deposition, alleviating its mismatch with the VBM of the Sn‐based perovskites (around −4.8 eV). However, the huge misalignment between the CBM of these perovskites and the lowest unoccupied molecular orbital energy level of C_60_ may be disadvantageous for obtaining a higher *V*_oc_. We believe that finding more suitable electron transport materials for Sn‐based PSCs should be one of the focuses in future research.

**Figure 3:**
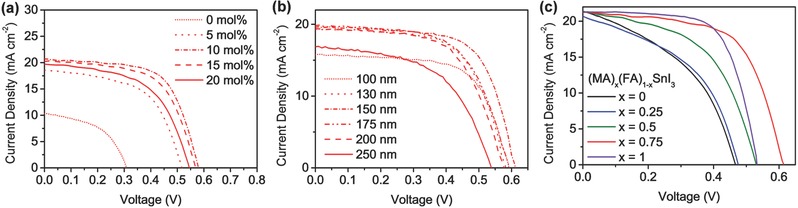
a) *J*–*V* curves of the devices based on (FA)_0.75_(MA)_0.25_SnI_3_ with different SnF2 molar ratios. b) *J*–*V* curves of the devices based on (FA)_0.75_(MA)_0.25_SnI_3_ with different perovskite layer thicknesses. c) *J*–*V* curves of the devices based on (FA)*_x_*(MA)_1_ *_−x_*SnI_3_ (*x* = 0.00, 0.25, 0.50, 0.75, and 1.00). All the curves were measured using forward scan mode at a scan rate of 300 mV s^−1^ under the simulation of AM 1.5G, 100 mW cm^−2^.

To elucidate the impact of the SnF_2_ concentration on device performance, we deposited (FA)_0.75_(MA)_0.25_SnI_3_ films using precursor solutions with different SnF_2_ molar ratios and fabricated corresponding devices. The photovoltaic parameters and the current density–voltage (*J*–*V*) curves of the devices are shown in Table S1 of the Supporting Information and **Figure** **3**a, respectively. As is depicted in the scanning electron microscope (SEM) images (Figure S4, Supporting Information), the SnF_2_‐free film shows the smallest grain size and has a high density of pinholes, resulting in a very poor average PCE of 1.31% ± 0.19%. As the SnF_2_ molar ratio increases, an enhancement of grain size can be observed. The film becomes pinhole‐free when the SnF_2_ amount reaches 10 mol%, and the corresponding device shows an optimal performance. However, with higher SnF_2_ amounts, phase separation becomes evident and pinholes reappear, resulting in a reduction in photovoltaic parameters. The influence of absorber layer thickness on device performance was also studied. As shown in Table S2 of the Supporting Information and Figure 3b, the devices with a 150 nm thick perovskite layer exhibit optimal performance. Therefore, we chose a SnF_2_ molar ratio of 10 mol% and an absorber layer thickness of 150 nm for later perovskite deposition and device fabrication.

We fabricated devices based on perovskites with different FAI/MAI ratios and measured their *J*–*V* curves accordingly under the simulation of AM 1.5G, 100 mW cm^−2^ under forward scan mode. The photovoltaic parameters are summarized in **Table** **1**. The performance of the MASnI_3_‐based device is relatively inferior, with an average PCE of 3.61% ± 0.32%. As the FA content increases, the *V*_oc_ and FF of the devices are gradually improved as compared to the MASnI_3_‐based devices, resulting in an enhancement in PCE. For the FASnI_3_‐based devices, the average FF is comparable to that of the (FA)_0.75_(MA)_0.25_SnI_3_‐based devices, while the average *V*_oc_ is reduced compared to the (FA)_0.50_(MA)_0.50_SnI_3_‐ and (FA)_0.75_(MA)_0.25_SnI_3_‐based devices, resulting in an average PCE of 5.93% ± 0.62%. The highest average PCE of 7.48% ± 0.52% was observed for the devices based on (FA)_0.75_(MA)_0.25_SnI_3_. The *J*–*V* curves of the champion devices are shown in Figure 3c. As the FA concentration is enhanced, the series resistance of the devices is gradually lowered, while their shunt resistance increases (estimated from the reciprocals of the slopes at the *V*_oc_ and *J*_sc_ points of the *J*–*V* curves, respectively).

**Table 1:** Photovoltaic parameters of (FA)*_x_*(MA)_1_ *_−x_*SnI_3_‐based devices

Perovskitea)		*V*_oc_ [V]	*J*_sc_ [mA cm^−2^]	FF (%)	PCE (%)
MASnI_3_	Champion	0.46	21.4	42.7	4.29
	Average	0.46 ± 0.03	20.0 ± 0.9	39.2 ± 4.0	3.61 ± 0.32
(FA)_0.25_(MA)_0.75_SnI_3_	Champion	0.48	20.7	45.2	4.49
	Average	0.47 ± 0.01	20.3 ± 0.4	44.3 ± 0.9	4.25 ± 0.15
(FA)_0.50_(MA)_0.50_SnI_3_	Champion	0.53	21.3	52.4	5.92
	Average	0.52 ± 0.01	19.9 ± 1.1	52.7 ± 4.1	5.43 ± 0.40
(FA)_0.75_(MA)_0.25_SnI_3_	Champion	0.61	21.2	62.7	8.12
	Average	0.58 ± 0.03	21.0 ± 0.5	61.9 ± 1.8	7.48 ± 0.52
FASnI_3_	Champion	0.48	21.3	64.6	6.60
	Average	0.48 ± 0.01	20.9 ± 0.4	58.8 ± 5.8	5.93 ± 0.62

^a)^ The statistical data in this table including average values and standard deviations are calculated from 12 separate devices for each perovskite composition.

**Figure 4:**
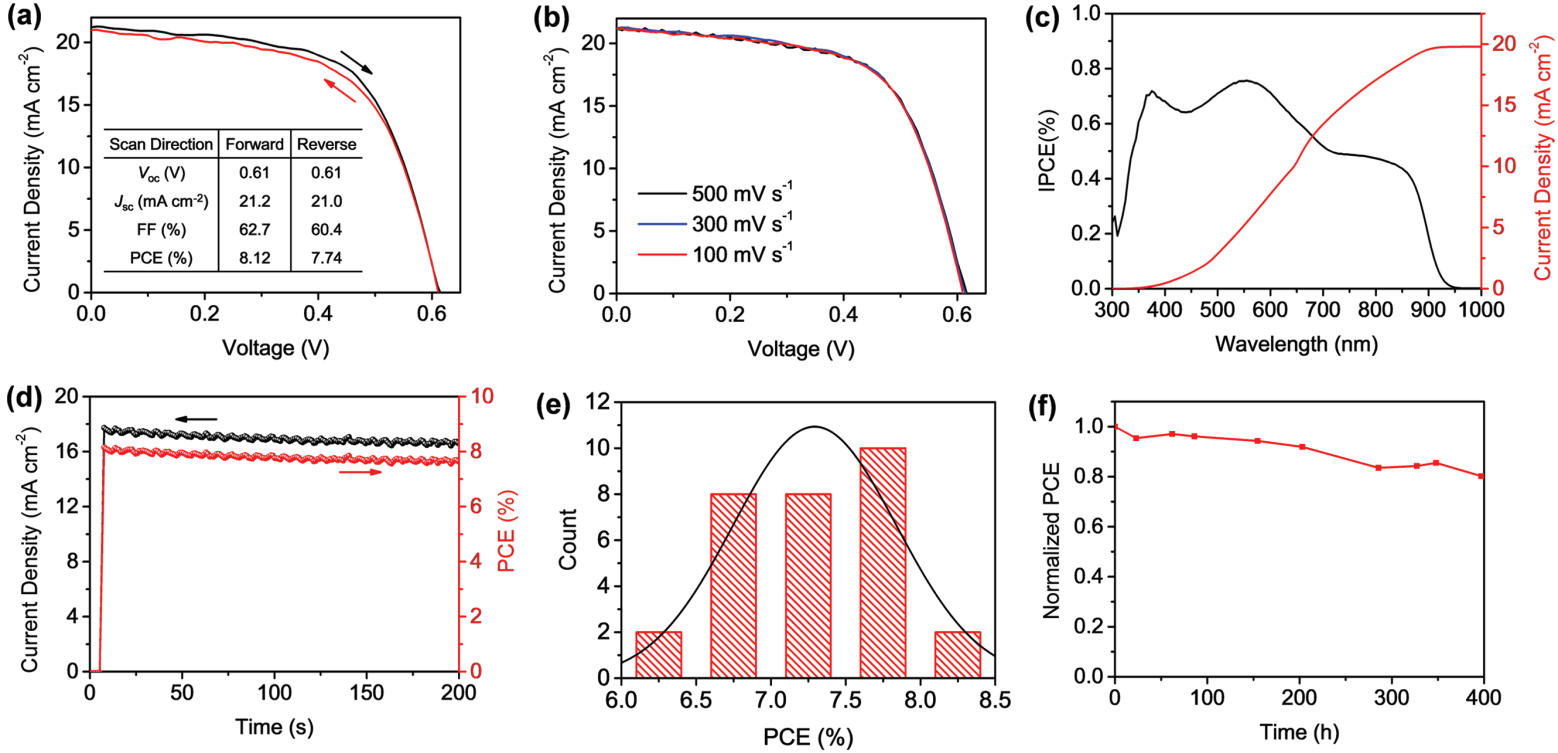
a) *J*–*V* curves of the champion device measured using both forward and reverse scan mode at a scan rate of 300 mV s^−1^ under the simulation of AM 1.5G, 100 mW cm^−2^. b) *J*–*V* curves of the champion device measured at different scan rates using forward scan mode under the simulation of AM 1.5G, 100 mW cm^−2^. c) IPCE spectrum of the encapsulated (FA)_0.75_(MA)_0.25_SnI_3_‐based device. d) Steady‐state *J*_sc_ and PCE of the (FA)_0.75_(MA)_0.25_SnI_3_‐based device measured at a bias of 0.46 V under AM 1.5G, 100 mW cm^−2^ irradiation. e) PCE histogram of 30 (FA)_0.75_(MA)_0.25_SnI_3_‐based devices from several fabrication batches. f) Normalized PCE of a (FA)_0.75_(MA)_0.25_SnI_3_‐based device stored in glovebox over a period of 400 h.

The champion (FA)_0.75_(MA)_0.25_SnI_3_‐based device exhibited a maximum PCE of 8.12% at forward scan, along with a *V*_oc_ of 0.61 V, a *J*_sc_ of 21.2 mA cm^−2^, and an FF of 62.7%. A small hysteresis is observed for the same device as it displays displayed a PCE of 7.74%, a *V*_oc_ of 0.61 V, a *J*_sc_ of 21.0 mA cm^−2^, and an FF of 60.4% at reverse scan (**Figure** **4**a). Also, the *J*–*V* curves measured at different scan rates show negligible differences (Figure 4b) and small hysteresis (Figure S5 and Table S3 in the Supporting Information). Furthermore, the integrated *J*_sc_ of the encapsulated (FA)_0.75_(MA)_0.25_SnI_3_‐based device obtained from the incident photon‐to‐electron conversion efficiency (IPCE) spectrum is 19.8 mA cm^−2^, which validates the *J*_sc_ obtained from *J*–*V* scans (Figure 4c). (In comparison, an integrated *J*_sc_ of 18.9 mA cm^−2^ was measured in air for the device without encapsulation, as shown in Figure S6 in the Supporting Information.) The best‐performing (FA)_0.75_(MA)_0.25_SnI_3_‐based device displays displayed a steady‐state photocurrent of ≈17.0 mA cm^−2^ over a period of 200 s at a bias of 0.46 V under 100 mW cm^−2^ AM 1.5G irradiation, which corresponds to a steady‐state output power of ≈7.8% (Figure 4d). We also fabricated 30 devices in several batches to testify the reproducibility of our (FA)_0.75_(MA)_0.25_SnI_3_‐based devices. As depicted in the PCE histogram in Figure 4e, the average PCE of the 30 devices is 7.29% ± 0.55%, with an average *V*_oc_ of 0.57 ± 0.02 V, an average *J*_sc_ of 20.7 ± 0.6 mA cm^−2^, and an average FF of 61.6% ± 2.5% under forward scan mode, revealing the high reproducibility of our device fabrication. Also, the (FA)_0.75_(MA)_0.25_SnI_3_‐based device stored in a nitrogen‐filled glovebox maintained ≈80% of its original PCE over a period of 400 h (Figure 4f).

**Figure 5:**
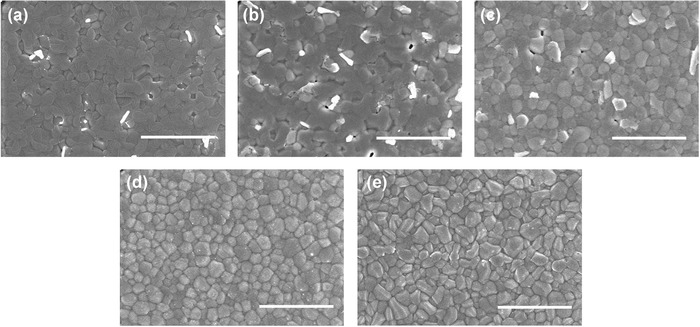
SEM images of a) MASnI_3_, b) (FA)_0.25_(MA)_0.75_SnI_3_, c) (FA)_0.50_(MA)_0.50_SnI_3_, d) (FA)_0.75_(MA)_0.25_SnI_3_, and e) FASnI_3_ films deposited on ITO/PEDOT:PSS substrates (scale bar: 3.0 µm).

To illustrate the impact of perovskite film quality on device performance, we examined the morphology of (FA)*_x_*(MA)_1_ *_−x_*SnI_3_ films using SEM (**Figure** **5**). The MASnI_3_ film exhibits a more continuous distribution of perovskites without much obvious grain boundaries. When the content of FA was enhanced, the grain boundaries gradually became more evident, and the FASnI_3_ film shows crystal grains with sharp edges and clear boundaries. At lower FA contents (*x* = 0.00, 0.25, and 0.50), some white grains are found in the films, which may be ascribed to the phase separation induced by SnF_2_.^19^, ^27^ These films also exhibit incomplete coverage with a few pinholes, which may explain the low FF and the slightly lower average *J*_sc_ of their corresponding devices. However, with higher FA contents (*x* = 0.75 and 1.00), the film morphology was greatly improved, displaying complete coverage and no evident phase separation, which may contribute to the superior device performance.

**Figure 6:**
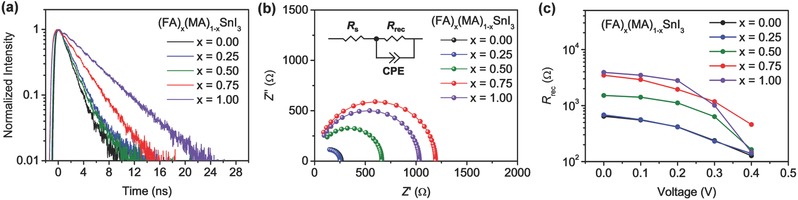
a) Time‐resolved spectra of (FA)*_x_*(MA)_1_ *_−x_*SnI_3_ (*x* = 0.00, 0.25, 0.50, 0.75, and 1.00) films deposited on quartz substrates. b) Nyquist plots of the devices based on (FA)*_x_*(MA)_1_ *_−x_*SnI_3_ (*x* = 0.00, 0.25, 0.50, 0.75, and 1.00) at 0.3 V under simulated 100 mW cm^−2^ AM 1.5G illumination measured at frequencies ranging from 100 000 to 100 Hz. Inset: the equivalent circuit model for fitting the plots. c) The fitted *R*_rec_ at different applied voltages obtained from the EIS analysis.

To further elucidate the role of cation mixing in inhibiting carrier recombination, the transient state PL spectra of the encapsulated perovskite films on quartz substrates were measured to compare the rate of carrier recombination in these perovskites (**Figure** **6**a). Using single exponential decay curve fitting, the PL lifetimes of the films are calculated to be 1.62 ± 0.01, 2.13 ± 0.01, 2.08 ± 0.02, 3.07 ± 0.01, and 4.92 ± 0.02 ns for x = 0.00, 0.25, 0.50, 0.75, and 1.00, respectively. The increasing trend in PL lifetime indicates that the incorporation of FA cations can effectively reduce carrier recombination in these films. In order to evaluate the extent of carrier recombination in the devices, we also carried out the electrochemical impedance spectroscopy (EIS) under simulated 100 mW cm^−2^ AM 1.5G illumination. The corresponding equivalent circuit of the devices is schemed in the inset of Figure 6b, which includes the series resistance (*R*_s_), recombination resistance (*R*_rec_), and constant phase element (CPE) of electrical double layer. For all the devices, the Nyquist plots display a main arc at low frequencies (Figure 6b), which can be attributed to the *R*_rec_ and CPE. The corresponding *R*_rec_ fitted from the Nyquist plots of the devices under different bias is shown in Figure 6c (the *R*_rec_ values are listed in Table S4 in the Supporting Information). At lower applied voltages, the *R*_rec_ increases as the FA content is enhanced, showing a decreasing trend in recombination rate that is similar to the results suggested by PL lifetime measurements. However, as the applied voltage is elevated, the *R*_rec_ of the FASnI_3_‐based device drops quickly, and at 0.4 V, it becomes substantially smaller than that of the (FA)_0.75_(MA)_0.25_SnI_3_‐based device and close to those of the MASnI_3_‐ and (FA)_0.25_(MA)_0.75_SnI_3_‐based devices. Since it is reported by Juarez‐Perez et al., larger *R*_rec_ at high applied voltages can contribute to higher *V*_oc_,^33^ we examined the *R*_rec_ at the high bias of 0.4 V and found it in good agreement with the *V*_oc_ of the devices. The (FA)_0.75_(MA)_0.25_SnI_3_‐based device has the largest *R*_rec_ at high applied voltages, which validates its outstanding *V*_oc_ as a result of the reduction of recombination rate through cation mixing.

## 3 Conclusion

In summary, we have employed a facile yet effective method of organic‐cation mixing to improve the efficiency of Sn‐based PSCs. The mixing of FA and MA cations leads to improvement in the film morphology of Sn‐based perovskites and reduction of charge carrier recombination in the solar cells. As a result, the PSCs based on (FA)_0.75_(MA)_0.25_SnI_3_ with 10 mol% SnF_2_ additive generated a maximum PCE of 8.12% and an average PCE of 7.48% ± 0.52%. The *V*_oc_ of our champion (FA)_0.75_(MA)_0.25_SnI_3_‐based device was enhanced to a remarkable 0.61 V, which outperforms most of the lead‐free PSCs that have been reported. Our work demonstrates that composition engineering is one of the important approaches to achieve higher *V*_oc_ and PCE of Sn‐based PSCs. We believe that our method, combined with more progress such as structure design and interface engineering, may further enhance the performance of Sn‐based PSCs.

## 4 Experimental Section

*Materials*: Formamidinium iodide (NH_2_CH=NH_2_I) and methylammonium iodide (CH_3_NH_3_I) were synthesized as previously reported.^34^, ^35^ ITO‐coated glass substrates (sheet resistance 8 Ω sq^−1^, thickness 180 nm) were obtained from Shenzhen Huayulianhe Co., Ltd. PEDOT:PSS (Clevious P VP AI 4083), SnI_2_ (99.999%), C_60_ (99.9%), BCP (99.9%), and Ag (99.99%) were purchased from H. C. Stark Company, Alfa Aesar, Puyang Yongxin Fullerene Technology Co., Ltd, Xi'@an Polymer Light Technology Corp., and China New Metal Materials Technology Co., Ltd., respectively.

*Perovskite Film Preparation*: The deposition of perovskite films was performed in a nitrogen‐purged glovebox (O_2_ and H_2_O concentrations kept below 1.0 and 0.02 ppm, respectively). The FASnI_3_/MASnI_3_ precursor solution was prepared by dissolving SnI_2_ (372 mg), SnF_2_ (16 mg), and FAI (172 mg)/MAI (159 mg) in dimethyl sulfoxide (1250 µL), and the (FA)*_x_*(MA)_1_ *_−x_*SnI_3_ (*x* = 0.25, 0.50, and 0.75) precursor solutions were prepared by mixing the FASnI_3_ and MASnI_3_ precursor solution at a volume ratio of *x*: (1 − *x*). The precursor solutions were filtered through a 0.25 µm filter and spin‐coated on substrates (PEDOT:PSS or quartz) at 5000 rpm for 90 s. Chlorobenzene was dripped onto the substrate at the 60th s during the spin‐coating process. All the perovskite films were annealed on a hotplate at 100 °C for 10 min.

*Device Fabrication*: The ITO‐coated glass substrates were cleaned successively with detergent, deionized water, acetone, and isopropyl alcohol in an ultrasonic bath for 20 min, respectively, and treated with oxygen plasma for 5 min. PEDOT:PSS aqueous solution was filtered through a 0.45 µm filter and spin‐coated on the ITO surface at 500 rpm for 9 s and 4000 rpm for 60 s, and then annealed at 140 °C for 20 min. The procedures afterward were performed in a nitrogen‐purged glovebox (O_2_ and H_2_O concentrations were kept below 1.0 and 0.02 ppm, respectively). The perovskite layer was deposited on the PEDOT:PSS substrates, and C_60_ (50 nm), BCP (8 nm), and Ag (100 nm) layers were sequentially deposited on the perovskite layer by thermal evaporation under vacuum (10^−6^ mbar) through a shadow mask with an active area of 0.10 cm^2^.

*Characterization*: The XRD patterns were obtained by a D/MAX‐2000 X‐ray diffractometer with monochromatic Cu Kα irradiation (λ = 1.5418 Å). The thickness of PEDOT:PSS and perovskite films was determined by a KLATencor α‐Step Surface Profiler. The optical absorption spectra were scanned with a Shimadzu UV–vis–NIR spectrometer (UV‐3600). The steady‐state PL spectra were obtained with an FLS980 fluorescence spectrometer (Edinburgh Instruments Ltd.) with an excitation wavelength of 490 nm. The ionization potentials were measured on an AC‐2 photoelectron spectrometer (Riken‐Keiki). The *J*–*V* curves of the solar cells were measured utilizing a Keithley 4200 semiconductor characterization system with an Oriel 300 W solar simulator (Thermo Oriel 91160‐1000) under simulated 100 mW cm^−2^ AM 1.5G irradiation in a nitrogen‐purged glovebox (O_2_ and H_2_O concentrations kept below 1.0 and 0.02 ppm, respectively), and the scan rate is set to 300 mV s^−1^ unless otherwise stated. The IPCE spectra were recorded on a Keithley 2400 sourcemeter under an irradiation of a 150 W tungsten lamp with a 1/4 m monochromator (Spectral Product DK 240). SEM was carried out on a Hitachi S‐4800 field emission SEM. The time‐resolved PL measurements were carried out by a Deltaflex TCSPC system (Horiba) at 950 nm with a 479 nm laser. The EIS measurements were performed on a CHI660 electrochemical workstation (CH Instrument Inc.) under simulated 100 mW cm^−2^ AM 1.5G irradiation.

## Conflict of Interest

The authors declare no conflict of interest.

## Supporting information

Supplementary
