# Supplementary material for: Mixed‐Organic‐Cation Tin Iodide for Lead‐Free Perovskite Solar Cells with an Efficiency of 8.12%
Source: Adv Sci (Weinh). 2017 Jul 14;4(11):1700204. doi: 10.1002/advs.201700204 (PMC5700647; doi:10.1002/advs.201700204)
Supplement: Supplementary file 1 — Supplementary [file ADVS-4-na-s001.pdf]

Copyright WILEY-VCH Verlag GmbH & Co. KGaA, 69469 Weinheim, Germany, 2017.

ADVANCED SCIENCE Open Access

Supporting Information

for *Adv. Sci.,* DOI: 10.1002/advs.201700204

Mixed-Organic-Cation Tin Iodide for Lead-Free Perovskite Solar Cells with an Efficiency of 8.12%

Ziran Zhao, Feidan Gu, Yunlong Li, Weihai Sun, Senyun Ye, Haixia Rao, Zhiwei Liu, Zuqiang Bian,* and Chunhui Huang*

Copyright WILEY-VCH Verlag GmbH & Co. KGaA, 69469 Weinheim, Germany, 2016.

Supporting Information

Mixed-organic-cation Tin Iodide for Lead-free Perovskite Solar Cells with an Efficiency of 8.12%

Ziran Zhao, Feidan Gu, Yunlong Li, Weihai Sun, Senyun Ye, Haixia Rao, Zhiwei Liu, Zuqiang Bian,* and Chunhui Huang*

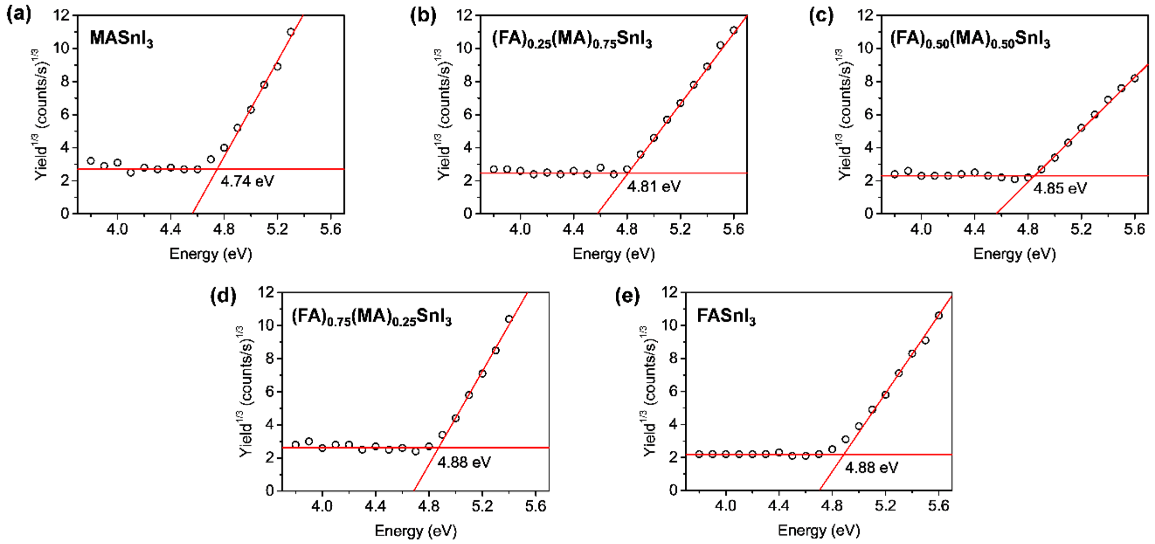

Figure S1. Photoemission spectroscopy measurements of (a) $MASnI_3$, (b) $(FA)_{0.25}(MA)_{0.75}SnI_3$, (c) $(FA)_{0.50}(MA)_{0.50}SnI_3$, (d) $(FA)_{0.75}(MA)_{0.25}SnI_3$, and (e) $FASnI_3$ films deposited on ITO/PEDOT:PSS substrates. The valence band maxima (VBM) were estimated to be 4.74, 4.81, 4.85, 4.88, and 4.88 eV for $MASnI_3$, $(FA)_{0.25}(MA)_{0.75}SnI_3$, $(FA)_{0.50}(MA)_{0.50}SnI_3$, $(FA)_{0.75}(MA)_{0.25}SnI_3$, and $FASnI_3$, respectively, from the intersections of the ground level lines and the regression lines.

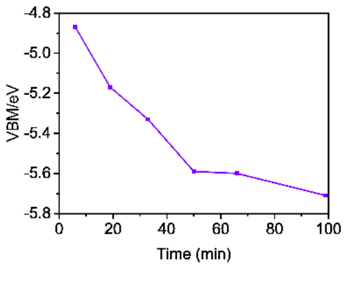

Figure S2. The valence band maxima (VBM) of $FASnI_3$ after different exposure time in air.

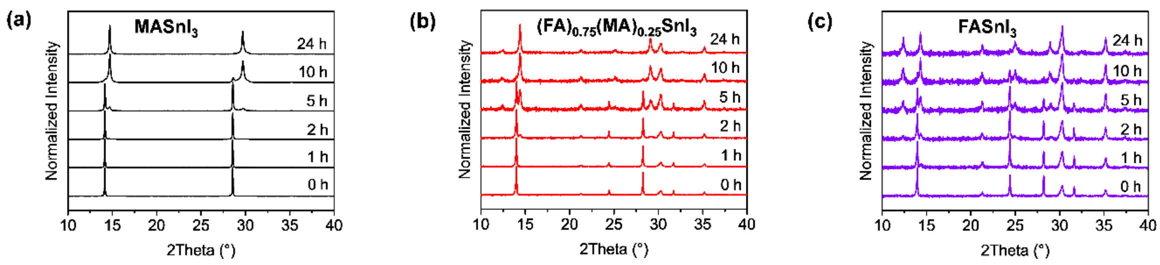

Figure S3. XRD patterns of (a) $MASnI_3$, (b) $(FA)_{0.75}(MA)_{0.25}SnI_3$, and (c) $FASnI_3$ films measured in ambient environment (temperature ~24 °C, relative humidity ~40%) for 24 h.

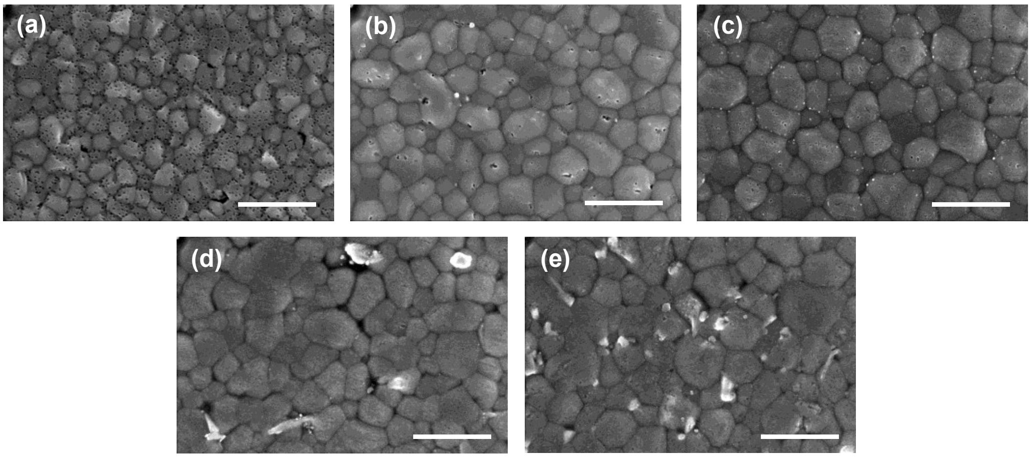

Figure S4. Scanning Electron Microscope (SEM) images of $(FA)_{0.75}(MA)_{0.25}SnI_3$ films prepared with (a) 0 mol% (b) 5 mol%, (c) 10 mol%, (d) 15 mol%, and (e) 20 mol% SnF_2 deposited on ITO/PEDOT:PSS substrates. Scale bar: 1.0 µm.

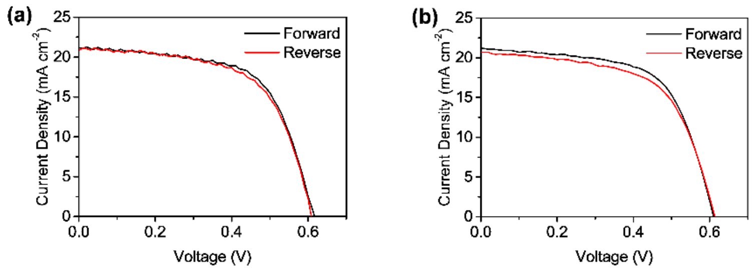

Figure S5. *J-V* curves of the champion device measured under the simulation of AM 1.5G, 100 mW cm^{-2} using forward and reverse scan modes at a scan rate of (a) 500 mV s^{-1} and (b) 100 mV s^{-1}.

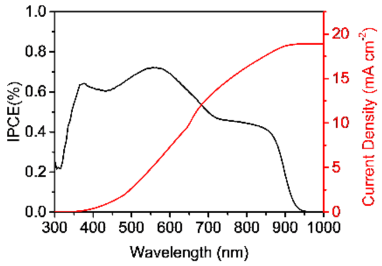

Figure S6. IPCE spectrum of the $(FA)_{0.75}(MA)_{0.25}SnI_3$-based device measured in air without encapsulation. The integrated J_{sc} is 18.9 mA cm^{-2}.

Table S1. Photovoltaic parameters of devices based on $(FA)_{0.75}(MA)_{0.25}SnI_3$ with different SnF_2 molar ratios[a)]

SnF_2 ratio		V_{oc} (V)	J_{sc} (mA cm^{-2})	FF (%)	PCE (%)
0 mol%	Champion	0.31	10.3	49.0	1.57
	Average	0.32 ± 0.01	8.8 ± 1.4	47.1 ± 1.2	1.31 ± 0.19
5 mol%	Champion	0.52	18.6	55.8	5.39
	Average	0.52 ± 0.00	18.4 ± 0.2	54.7 ± 0.8	5.24 ± 0.09
10 mol%	Champion	0.58	20.7	63.2	7.59
	Average	0.58 ± 0.01	20.6 ± 0.2	61.2 ± 1.8	7.28 ± 0.17
15 mol%	Champion	0.55	20.2	60.6	6.98
	Average	0.56 ± 0.01	20.6 ± 0.4	57.1 ± 2.1	6.59 ± 0.22
20 mol%	Champion	0.55	19.7	54.8	5.94
	Average	0.56 ± 0.01	19.0 ± 0.4	57.9 ± 1.1	5.33 ± 0.40

[a)]The statistical data including average values and standard deviations are calculated from 6 separate devices for each SnF_2 molar ratio.

Table S2. Photovoltaic parameters of $(FA)_{0.75}(MA)_{0.25}SnI_3$-based devices with different perovskite layer thicknesses[a)]

Thickness (nm)		V_{oc} (V)	J_{sc} (mA cm^{-2})	FF (%)	PCE (%)
100	Champion	0.59	15.8	66.4	6.18
	Average	0.58 ± 0.01	15.7 ± 0.2	62.6 ± 4.5	5.73 ± 0.46
130	Champion	0.58	19.3	64.0	7.16
	Average	0.57 ± 0.02	18.5 ± 0.6	62.0 ± 1.9	6.48 ± 0.44
150	Champion	0.62	19.6	67.7	8.10
	Average	0.61 ± 0.01	19.8 ± 0.3	64.0 ± 2.2	7.68 ± 0.27
175	Champion	0.59	19.8	62.1	7.26
	Average	0.58 ± 0.01	18.6 ± 0.8	59.7 ± 2.1	6.50 ± 0.41
200	Champion	0.58	19.4	61.1	6.88
	Average	0.58 ± 0.01	18.7 ± 0.9	60.0 ± 4.2	6.45 ± 0.43
250	Champion	0.54	16.9	54.6	4.98
	Average	0.54 ± 0.01	16.0 ± 0.5	53.6 ± 3.0	4.63 ± 0.26

[a)]The statistical data including average values and standard deviations are calculated from at least 10 separate devices for each perovskite layer thickness.

Table S3. Photovoltaic parameters of the champion device with different scan rates and scan directions

Scan rate (mV s^{-1})		V_{oc} (V)	J_{sc} (mA cm^{-2})	FF (%)	PCE (%)
500	Forward	0.62	21.2	61.6	8.09
	Reverse	0.61	20.9	61.7	7.85
300	Forward	0.61	21.2	62.7	8.12
	Reverse	0.61	21.0	60.4	7.74
100	Forward	0.61	21.2	62.2	8.03
	Reverse	0.61	20.7	60.4	7.63

Table S4. Recombination resistances (R_{rec}) of $(FA)_x(MA)_{1-x}SnI_3$-based devices fitted from the Nyquist plots

Bias (V)	x = 0	x = 0.25	x = 0.50	x = 0.75	x = 1
0	645.1	680.3	1516	3479	3917
0.1	552.9	560.6	1413	2907	3488
0.2	416.5	414.2	1115	1946	2797
0.3	236.9	230.8	634.3	1177	1010
0.4	127.3	138.9	163.6	459.2	146.5
